# Modified Benelli procedure for subcutaneous mastectomy in gynecomastia: A randomised controlled trial

**DOI:** 10.1016/j.amsu.2019.09.007

**Published:** 2019-09-18

**Authors:** Riyadh Mohamad Hasan

**Affiliations:** University of Baghdad, Al-Kindy College of Medicine, Department of Surgery, Iraq

**Keywords:** Gynecomastia, Benelli, Surgery

## Abstract

**Background:**

Gynecomastia is defined as a benign glandular proliferation of the male breast tissue causing enlargement of the breast and a feminine appearance. Gynecomastia is usually treated surgically in some patients by different techniques.

**Aim of the study:**

is to allow ample excess during excision and to remove excess skin to allow for better cosmetic results using “modified Benelli technique” and to obtain good breast shape with better nipple areola complex position without any breast tension.

**Type of the study:**

Randomised controlled trial study.

**Patients and methods:**

The study included 150 patients with gynecomastia (Grade II and III) for the period between January 2010 and January 2016 who attended private hospitals and Al-Kindy Teaching Hospital. The patients were divided into two groups according to the operative techniques used. Group A included 75 patients treated surgically with subcutaneous mastectomy using periareolar incision. Group B; included the other 75 patients who were managed by “modified Benelli technique".

**Results:**

The subcutaneous mastectomy using “modified Benelli technique” showed a significantly lower operating time due to ample access for excision of breast tissue. Excision of excess skin allowed the areola to retain a cosmetically more acceptable position. There was a lot of pleating of the skin compared to the other technique using the periareolar incision.

**Conclusions:**

This technique namely the “modified Benilli technique” provides a relatively simple method with an aesthetically good outcome to treat gynecomastia with a low rate of complications and recurrences.

## Highlights

• Gynecomastia is defined as a benign glandular proliferation of the male breast tissue.
• Is to allow ample excess during excision and to remove excess skin to allow for better cosmetic results using “modified Benelli technique".
• Excision of excess skin allowed the areola to retain an acceptable position, with better cosmetic shape of areola.
• This technique namely the “modified Benilli technique” provides a relatively simple method with an aesthetically good outcome.

## 1 Introduction

Gynecomastia is a generalized enlargement of male breast tissues. Most cases are idiopathic especially during puberty and the pathophysiological mechanism involves various diseases including hepatic disease, renal diseases, and endocrine diseases [1].Gynecomastia has an overall incidence of 32–40%, with a prevalence reported as high as 65% in elderly males [2].True gynecomastia is characterized by hypertrophy of the stroma and glandular tissue, while pesudogynecomastia results from obesity and adipose tissue hypertrophy [3,4]. Based on skin elasticity, the presence of an inframamary fold and mammary ptosis or drooping, Cordova and Moschella classified Gynecomastia into four grades of increasing severity ranging from simple areolar protrusion, to female breasts shape depend on the relationship between the nipple-areola complex and the inframamary fold [5,6]. Treatment of gynecomastia is self-limiting in puberty and fibrous tissue replaces the proliferation of glandular tissue [7] but if it persists and be associated with pain, tenderness and psychological upset, surgical and medical treatment must be started [8]. The goal of surgery is to restore the normal appearance of male thorax with little scar depending on degree of gynecomastia [9]. There are a lot of other options now adays for treatment like subcutaneous mastectomy that involves direct resection of the glandular tissue using a peri-areolar or trans-areolar approach with or without liposuction [10]. These methods were associated with many complications like contour irregularity of the breast, hematoma, seroma, numbness, necrosis, breast asymmetry, and cosmetic non satisfaction [11].According to the stages of the gynecomastia and the cosmetic preference many types of incisions were used to provide optimal results [12].Regarding females’ cosmetic operation for the breast, “around the Block” incision was used by Benelli for ptosis of the breast and returning the nipple areola complex to a more acceptable position. However, it resulted in pleating of the suture line, but these pleats were reported to disappear with time [13]. This study was conducted to evaluate the efficacy of a modified approach to that used by Benelli (modified Benelli technique), especially in the treatment of stage II and III gynecomastia.

## 2 Patients and methods

Patients with true gynecomastia, attending Al-Kindy Teaching Hospital and private hospitals, Baghdad, Iraq, from January 2010 to January 2016 were enrolled in the study. Grading of gynecomastia was done according to Cordova and Moschella classification [6]. Patients with Grade I were excluded because of no skin excess, while grade IV were excluded due to the need of more extensive procedures. The study protocol was reviewed and approved by the Scientific and Ethical Committee of Al-Kindy College of medicine and Al-kindy Teaching hospital in the committee number 6 in 20-5-2019 (Ethical approval number and Date). Written informed consents were obtained from all patients as an action on acceptance. All enrolled patients were operated by the same surgeon. Patients were randomly assigned to one of the following surgical technique:

Group A; included patients who underwent subcutaneous mastectomy using periareolar incision with lateral and medial extension as needed according to Webster [12].

**Fig. 1:**
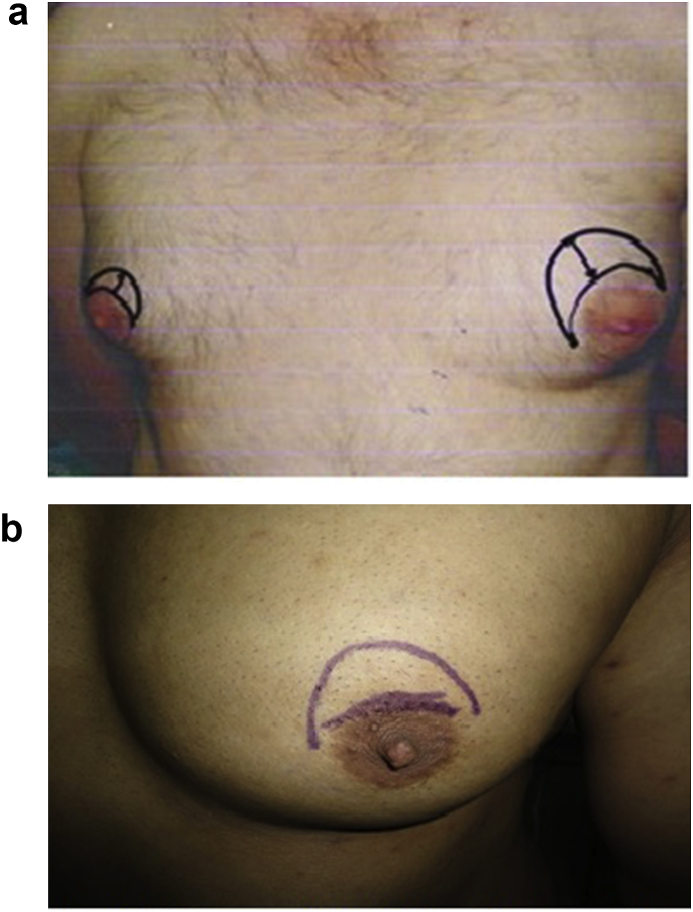
(a) The proposed Modified Benelli (MBT) showing the point X and lines A and B. (b) The proposed Modified Benelli Technique (MBT).

Group B; included patients who were treated by the proposed Modified Benelli Technique (MBT) using the following operative procedure:

First ask the patient to stand up and draw the a line referrin to the midline of the chest then mark the ideal nipple areola complex at 18 cm from midclavicular point and about 20 cm from the sternal notch and about 11 cm from midsternal line according to Beckenstein and colleagues [14]. This point will determine the position where the nipple should be placed (point X) with skin marker pen and assess the quantity of surplus skin to be excised. After that, ask the patient to lie down on the couch and a periareolar line was marked (line A) above and medial to the areola and a second radial line above it and parallel to it passing in the point (X) was made and named line B. The ends of this line is curved to approximate and connect to both ends of the line A as illustrated in Fig. 1a and b. Then, the patient is given general anesthesia and the two incisions were made on the lines A and B i.e. periareolar incision above and medial to the areola with a second incision above it and connecting both ends. Next, the whole thickness of the excess skin between line A and the line B was excised (Simon classification 2A, 2B and 3) and subcutaneous mastectomy was done and sent to histopathology. Later on, bleeding control was done by good hemostasis and suction drain was put in its proper site. Finally subcuticular suturing was done by approximation of two incisions using Nylon 3/0. Lastly, sterile pressure dressing was placed.

The result of both groups were compared in terms of operating time, nipple-areola complex location, post-operative complications including, pleating of the skin at suture line, hematoma, bruising at the site of incision, soft tissue deformity, seroma, hyposthesia of nipple-areolar complex, wound dehiscence, areolar epidermolysis, and hypertrophic scarring.

Regarding follow-up, the patients were appointed after 10 days (the day of removal of stitches) for the first postoperative visit and after 3 and 6 months for the second and third follow-up visits.The work has been reported in line with the STROCSS criteria [15]. The work is fully compliant with the CONSORT criteria (http://www.journal-surgery.net/article/S1743-9191%2811%2900565-fulltext) [16,17]. The work submitted with a clinical trail gov. com Registry UIN: www.clinical trail gov.com. Registration UIN of this work is NCT04063722.

**Statistical analysis:** Data expressed as mean ± Standard error mean. Student t-test was done to assess the level of significance. In addition to that percentages were used to express the frequency of some data and Chi^2^ was done to assess the level of significance. This was performed using Mini tab version13. P value less than 0.05 is considered statistically significant.

## 3 Results

A total of 150 patients with stage II and III gynecomastia were enrolled in the present study of which 75 patients were randomly assigned to each of the study groups. Differences in the clinical characteristics of the two study groups’ patients were not statistically significant as presented in Table 1.

**Table 1:** clinical characteristics of the patients.

Parameter	Group A		Group B		P –value
	75 patients		75 patients		
	No. (%)		No. (%)		
Type of Operation	subcutaneous mastectomy using periareolar incision (Jerome Webster)		subcutaneous mastectomy using “modified Benelli technique"		–
Age -years	25.6 ± 10.1		27.5 ± 9.5		0.891
BMI- kg/m^2^	25.21 ± 2.15		24.51 ± 3.21		0.554
Active smoking	56	74%	52	69.33%	0.583
Unilateral gynecomastia	50	66.66%	45	60%	0.497
Bilateral gynecomastia	25	33.33%	30	40%	0.497
Duration of gynecomastia	6 months −10 years		6 months-9 years		–
Testes volume					
Atrophied	5	6.7%	4	5.3%	0.325
Normal	70	93.3%	71	94.7%	0.424
Normal external sexual character	75	100%	75	100%	1
History of breast trauma	0	(0.0)%	0	(0.0)%	1
Breast consistency					
tender	6	8.0%	4	5.3%	0.32
non tender	69	92.0%	71	94.7%	0.43

In the majority of patients the cause of gynecomastia couldn't be identified (35; 46.4% in group A and 46; 61.3% in group B). Causes in the remaining patients are presented in Table 2, moreover, no significant differences were found as comparing group A and B.

**Table 2:** The underlying causes of gynecomastia.

Causes	Group A		Group B		P –value
	no	%	no	%	
Endocrine causes	15	20%	12	16%	0.671
drug induced	15	20%	12	16%	0.671
Metabolic cause	1013.3%		5	6.7%	0.275
Idiopathic	3546.7%	4661.3%			0.101

Unilateral gynecomastia was present in 50 patients (66.7%) in group A and in45 patients (60%) in group B, while bilateral gynecomastia was present in 25 patients (33.3%) and 30 patients (40%) of group B. accordingly the number of operations done by periareolar incision amounts to 100 and those operated by “modified Benelli technique” was 105.

Grade II gynecomastia was present in 22 patients (22%) and 5 patients (4.7%) in group A and B respectively while grade III was present in 78 patients (78%) and in100 patients (95.2%) of group A and B respectively as shown in Table 3.

**Table 3:** Preoperative grading of gynecomastia.

Grades	Group A No. of operation = 100 No. %		Group B No. of operation = 105 No. %		P –value
II	22	22	5	4.76	0.0006
III	78	78	100	95.2	

**Fig. 2:**
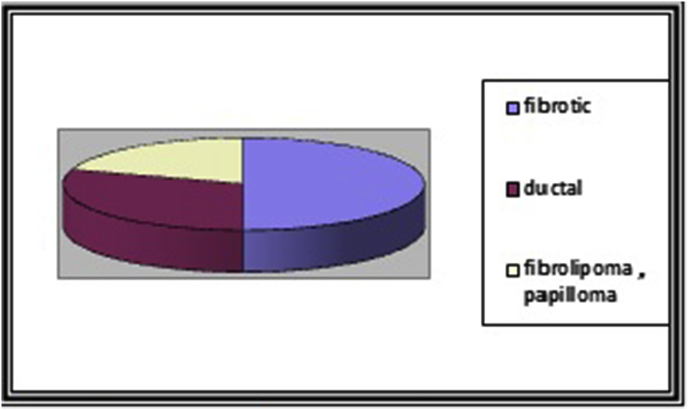
causes of gynecomastia.

The most common histopathological finding was fibrotic gynecomastia (50%), followed by ductal hyperplasia (30%) and breast papilloma (20%) Fig. 2.

**Fig. 3:**
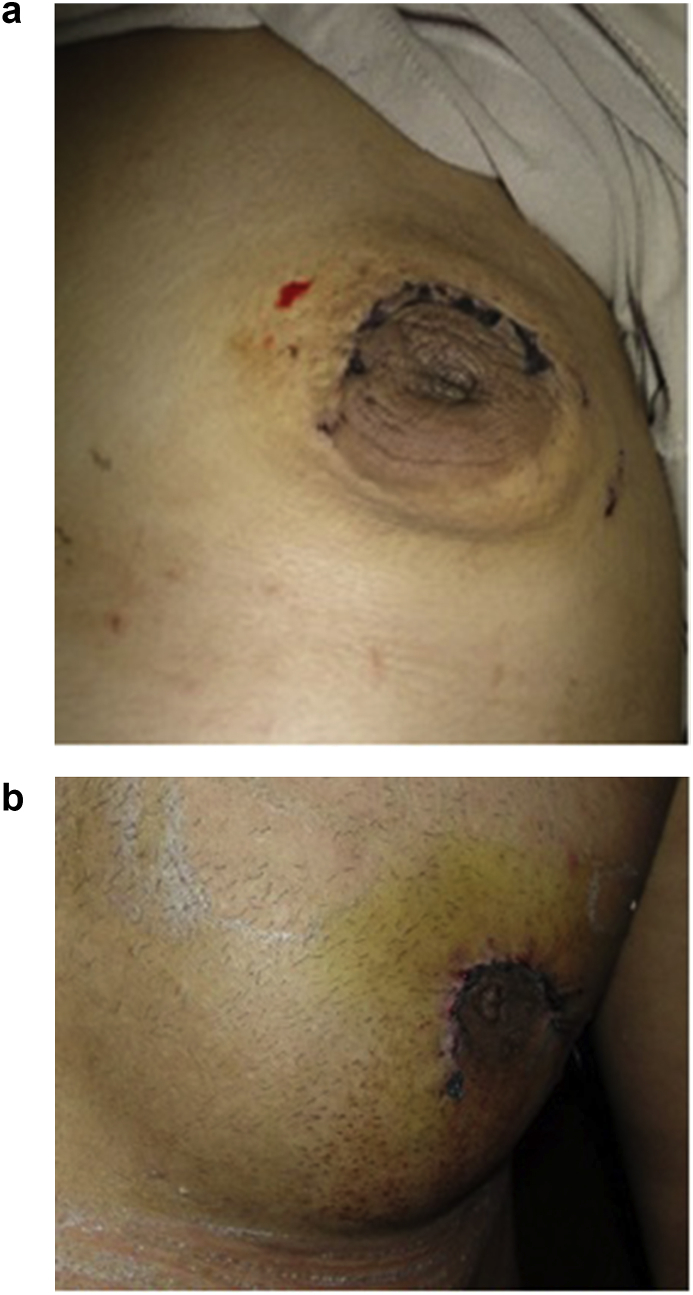
(a) Showing immediate posterative pleating at the suture line. (b) Showing immediate posterative pleating at the suture line.

**Fig. 4:**
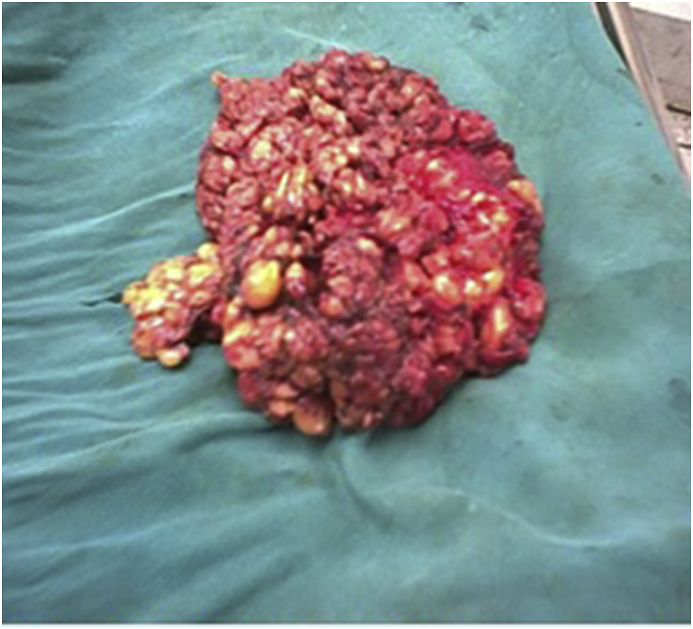
Glandular tissue that was removed.

**Fig. 5:**
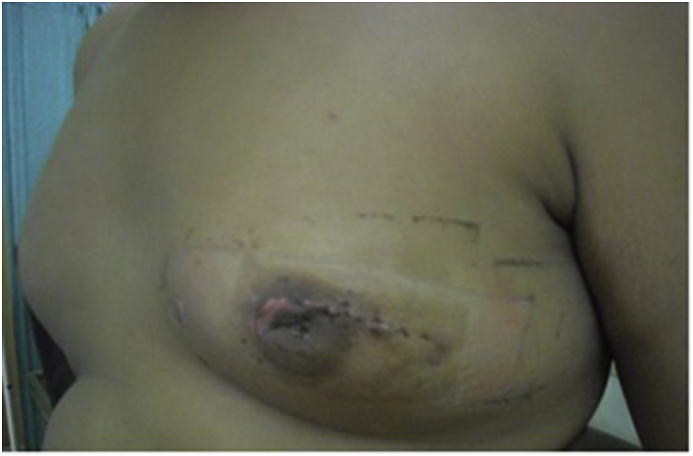
Follow-up the patient after 10 days.

**Fig. 6:**
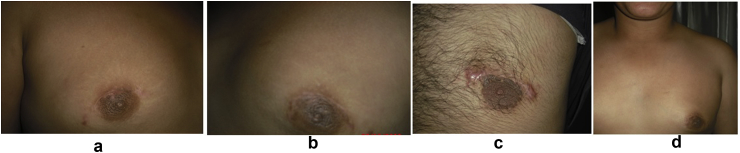
a,b,c,d showing the post operative scar results

Considering assessment of both techniques as detailed in Table 4, as compared to group A,subcutaneous mastectomy using"modified Benelli technique” (group B) showed a significantly shorter operating time, and more acceptable position with a better cosmetic shape of the nipple areola complex however this was associated with a higher rate of pleating at the suture line as demonstrated in Fig. 3a and b which showed immediate postoperative pleating at the suture line. The glandular tissue was excised as demonstrated in Fig. 4-.Then the patients were followed up after 10 days of operation (time of stich removal) as shown in Fig. 5- for the presence of hematoma or seroma and bruising; patients were also examined after 3 and 6 months to assess the post operative results regarding acceptable position of nipple areola complex, remote result of pleating of skin at suture line, hypertophic scaring and persistence of nipple hyposthesia (Fig. 6- a,b,c,d). there was a significantly increased rates of postoperative complications including hematoma, bruising and soft tissue deformity observed in group A as compared to group B. while there was no significant difference regarding occurrence of seroma, persistence of nipple hyposthesia, wound dehesceince, areolar epidermolysis or hypertrophic scarring.

**Table 4:** Assessment of subcutaneous mastectomy using different techniques in treatment of gynecomastia after 10 days and 3 months and 6 months postoperatively.

Parameters	Group A		Group B		p- value
	No. (%)		No. (%)		
No. of operations	100		105		
Mean Operating time (minutes)	45.2 ± 1.3		30.3 ± 2.4		0.0001
Acceptable position of nipple areola complex post operatively	22	22	71	67.61	0.0001
Pleating of the skin at suture line	5	5	70	66.66	0.0001
Hematoma formation	31	31	9	8.57	0.0001
Bruising at the site of incision	20	20	3	2.85	0.0002
Soft tissue deformity	60	60	20	9.04	0.0001
Seroma	17	17	12	11.42	0.345
Persistence of nipple hyposthesia	10	10	14	13.33	0.596
Wound dehiscence	3	3	5	4.76	0.777
Areolar epidermolysis	9	9	6	5.71	0.063
Hypertrophic scarring	2	2	1	0.95	1

The results of both groups were compared in terms of operating time, nipple-areola complex location, post-operative complications including, pleating of the skin at suture line, hematoma, bruising at the site of incision, soft tissue deformity, seroma, hyposthesia of nipple-areolar complex, wound dehiscence, areolar epidermolysis, and hypertrophic scarring after 10 days and 3 months and 6 months postoperatively.

## 4 Discussion

The mainstay in treatment of gynecomastia is direct periareolar incision and skin excision due to its simplicity and evading of extra instrumentation [18,19]. Moreover, Liposuction and ultrasound assisted mastectomy have been used in treatment of gynecomastia [20]. These methods had limited ability to deal with significant skin excess and ptosis but had minimal scar burden [21]. For mild to moderate gynecomastia, endoscopic techniques may be used [22] while severe cases required breast amputation with free nipple grafting [23]. The treatment of gynecomastia needs an individualized approach to provide sufficient resection of the skin and optimize aesthetic results [24]. The simple periareolar incision with lateral and medial extensions was used by Webester [12] but it failed to eliminate the excess skin in stage II and III. Inframamary, transareolar and other types of incisions have been devised for removal of the excess skin to returns the nipple areola complex to a more acceptable position anatomically, but unfortunately, it results in long relatively conspicuous scar (12).The significantly improved surgical outcome revealed in the present study with the new “modified Benilli technique”, as compared to the traditional periareolar incision in terms of a shorter operating time and lesser rate of hematoma formation can be attributed to the ample access to the underlying breast tissue which provided a better contact to bleeding points and better hemostasis in addition to simplifying the subcutaneous mastectomy. The more appropriate post-operative final shaping of the breast in group B as compared to group A is mainly due to removal of the excess skin giving a more acceptable position of the nipple areola complex.On the other hand the higher rate of pleating at the suture line could be due to the longer outer than inner incisions, However all pleats in the resulting suture line disappeared within about 6–8 months which is in agreement with the results reported by Benneli (13).The lower acceptability of post operative position of nipple areola complex observed in group A patients of the present study as compared to the results reported by Webster, could be attributed to the inclusion of stage II and III and the subsequent requirements of excess skin removal, although it should be stressed here that Webster had not reported the stages of the patients enrolled in his study. On the other hand the lesser retraction required in group B due the wide exposure may explain the fewer occurrence of traumatic retraction and the subsequent postoperative bruising. The pros of this method are wider operation field, excision of the tissue is easier, hemostasis is better so hematoma and seroma are less frequent, extensive retraction is not needed which lessens the operative trauma and the nipple will assume better cosmetic position due to removal of extra skin. On the other hand, the only cons is skin pleating which occurs at the site of skin incision that disappear on remote follow-up as shown in our study.

## 5 Conclusions

The technique proposed in the present study provides a relatively simple method with an aesthetically pleasing method to treat gynecomastia with a lower rate of complications as compared to the traditional techniques.

## Ethical approval

Al-Kindy college of medicine committee No. 6. (20-May-2019).

## Sources of funding

None.

## Author contribution

Dr Riyadh Mohamad Hasan did the operations and wrote the paper.

## Conflicts of interest

None.

## Trial registry number

1. Name of the registry: clinical trial gov. com
2. Unique Identifying number or registration ID: NCT04063722
3. Hyperlink to the registration (must be publicly accessible):

https://clinicaltrials.gov/ct2/results?cond=&term=NCT04063722&cntry=&state=&city=&dist=

## Guarantor

Prof Dr Batool Mutar Mahdi.

## Consent

None.

## Provenance and peer review

Not commissioned, externally peer reviewed.
